# Hippocampal-Temporopolar Connectivity Contributes to Episodic Simulation During Social Cognition

**DOI:** 10.1038/s41598-018-24557-y

**Published:** 2018-06-20

**Authors:** Corinna Pehrs, Jamil Zaki, Liila Taruffi, Lars Kuchinke, Stefan Koelsch

**Affiliations:** 1 Department of Psychology, Technische Universität Dresden, 01062 Dresden, Germany; 2 Bernstein Center for Computational Neuroscience, Charité – Universitätsmedizin, 10117 Berlin, Germany; 3 Department of Psychology, Northwestern University, Evanston, IL 60208 USA; 4 Department of Psychology, Stanford University, Stanford, CA 94305 USA; 5 Department of Education and Psychology, Freie Universität Berlin, 14195 Berlin, Germany; 6 International Psychoanalytic University, 10555 Berlin, Germany; 7 Department of Biological an Medial Psychology, University of Bergen, 5009 Bergen, Norway

## Abstract

People are better able to empathize with others when they are given information concerning the context driving that person’s experiences. This suggests that people draw on prior memories when empathizing, but the mechanisms underlying this connection remain largely unexplored. The present study investigates how variations in episodic information shape the emotional response towards a movie character. Episodic information is either absent or provided by a written context preceding empathic film clips. It was shown that sad context information increases empathic concern for a movie character. This was tracked by neural activity in the temporal pole (TP) and anterior hippocampus (aHP). Dynamic causal modeling with Bayesian Model Selection has shown that context changes the effective connectivity from left aHP to the right TP. The same crossed-hemispheric coupling was found during rest, when people are left to their own thoughts. We conclude that (i) that the integration of episodic memory also supports the specific case of integrating context into empathic judgments, (ii) the right TP supports emotion processing by integrating episodic memory into empathic inferences, and (iii) lateral integration is a key process for episodic simulation during rest and during task. We propose that a disruption of the mechanism may underlie empathy deficits in clinical conditions, such as autism spectrum disorder.

## Introduction

Memory powerfully contributes to social experience^1^. For instance, people connect with others’ emotions most easily when they are given background information about the situations that create those emotions^2,3^. *Episodic simulation* describes the use of episodic memories to understand current, past or future scenarios and to infer other people’s mental state^4^. Episodic simulation can increase prosocial behavior^5^, is impaired in hippocampal amnesia and dementias^6,7^ and declines with age^8^. The role of episodic mechanisms in promoting empathic concern, however, still remains unclear.

Episodic simulation is one type of memory processing that requires the integration of different memory types, namely episodic with semantic memory. Semantic memory describes context-independent general knowledge, which is necessary to understanding others’ emotions^9^. For instance, in drawing inferences about a friend who breaks up with their partner and later eats ice cream, one might draw on the semantic knowledge that breakups and ice cream generally produce negative and positive affect. Episodic memory, by contrast, describes context-dependent knowledge, which is declarative and is provided, for example, by context information to someone’s situation^10,11^. When people engage in episodic simulation, they integrate episodic with semantic memory. For instance, consider a perceiver learning about a social “target’s” painful breakup. The perceiver would likely combine this episodic context information with semantic information from current perceptual input (i.e. eating ice cream), which might further aid the perceiver in empathizing appropriately with the target. Mnemonic integration is thus a key process underlying episodic simulation.

Semantic and episodic memory systems are closely linked^12,13^. They share neural activations but are also mediated by distinct neural systems. They both engage the default mode network (DMN)^14,15^, a set of brain regions typically active during resting state compared with task state^16^. Key regions of the extended DMN show distinct selectivity for different memory systems^12,17^. The medial temporal lobe complex including the hippocampus (HP), for example, plays a central role in supporting episodic encoding and retrieval^18,19^. The temporal pole (TP), by contrast, is critically involved in semantic memory^20,21^ as underscored by studies on patients with neurodegenerative atrophy exhibiting severe deficits in semantic knowledge but spared episodic memory^22,23^. TP plays a prominent role in forms of inferences that require semantic processing, including processing of language^24^, faces^25^, social concepts^26^, empathic behavior^27^, and emotion^28^. Both, HP and TP, share a brain network for cognitive function requiring the use of episodic information in a constructive manner, like autobiographical memory, theory of mind, prospective thinking and the default mode^29,30^.

Frith and Frith^31^ have stated that TP’s role in mentalizing is to retrieve social knowledge on the basis of past experiences as a semantic frame for current perceptual input. One explanation for the involvement of TP in socio-emotional processing might be that its function is to integrate episodic with semantic memories.

As outlined above, there is extensive knowledge about how mnemonic integration works in the brain, including for emotional memory. However, little is known about how episodic simulation shapes empathic concern.

We here take a connectivity perspective to better understand (i) mnemonic integration, and (ii) its role in generating empathic concern based on complex cues. We expected that mnemonic integration would be implemented through connectivity between brain regions that are associated with different memory types, specifically TP and HP. This assumption was based on the literature^17,32^, but also on the findings of our previous study. In this study, participants viewed empathy-inducing film clips featuring a character experiencing emotions while they were scanned with fMRI. The film clips were either presented without a contextual framing, or with a neutral or sad contextual framing to provide background information about the character’s situation. The participants were instructed to always refer the presented context information to the situation of the character in the film clip. The context information provided episodic information and was integrated with the visual semantic input during film clips presentation. Even though no explicit memory test was conducted, the study design assures that participants used mnemonic processes to integrate prior context information and to episodically simulate the mind of the movie character.

**Figure 1:**
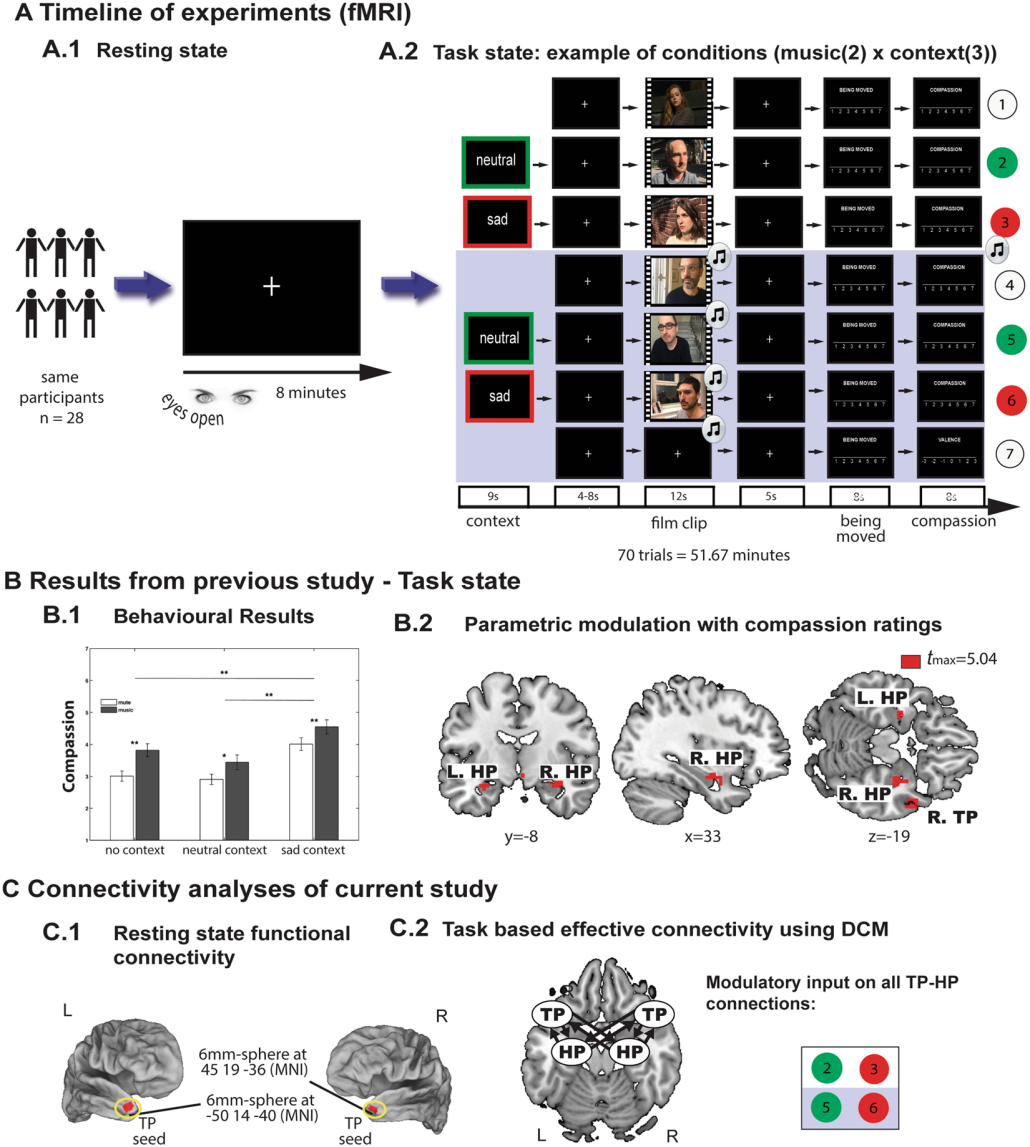
Timeline and design of experiments, results from previous study and outline of connectivity analyses. (**A**) The same participants (i.e. Berlin sample) underwent the measurement of fMRI data during 8 min of resting state (fixation cross, eyes open) [A.1], followed by 51 min 67 s of a social cognition task [A.2], in which the presentation of empathic film clips was varied by written context information preceding the film clips and music (blue background). The context information was either neutral (green) or sad (red). After the film clips the participants rated their emotional experience in terms of being moved and compassion on two 7-point Likert-scales (1 not at all, 7 very much). Example of video frames similar to those employed in the study. A fully randomized design was employed to present 70 trials in total (10 trials per condition). Source: Elena Panouli, Kai Görgen, Corinna Pehrs; photographers. Adapted with permission of the actresses, actors and photographers. (**B**) Previous analyses of behavioral task data have shown that music and sad context significantly increased the compassion ratings (error bars represent standard error of the mean *p < 0.05, **p < 0.001) [B.1]. A parametric modulation with compassion ratings revealed activations in bilateral anterior HP and TP [B.2] suggesting that an increase of empathic concern associated with sad context information is tracked by activity in these regions (cf. Pehrs *et al*.^2^). (**C**) To investigate the contribution of TP-HP connectivity to episodic simulation, a functional connectivity analysis was applied to resting state data [C.1] by looking for correlations of the fMRI time series of bilateral TP with each voxel in the rest of the brain. TP-HP effective connectivity was examined during task using dynamic causal modeling (DCM) [C.2]. This analysis tested the hypothesis that TP-HP connectivity changes as a function of context information and episodic simulation (for details on the Modelspace see Fig. 2). TP: temporal pole; HP: hippocampus, L: left, R: right, MNI: montreal neurological institute. Brain templates are taken from the Caret5 software.

We found that the participant’s empathic concern for a movie character was significantly increased by a sad compared to a neutral contextual framing (Fig. 1B.1). This increase of experienced empathic concern based on sad context information was in turn tracked by activity in the TP and anterior HP (aHP) as revealed by a parametric modulation analysis^2^ (p < 0.001 uncorrected, cluster extent > 5, Fig. 1B.2). The present study uses the same data and the same task of the previous study, but asks how the connectivity between TP and aHP changes as a function of context information that was shown to increase compassion ratings towards the movie character.

In addition, the connectivity between the TP and the HP was investigated during resting state using the same set of participants. This was done because mounting evidence shows that people engage in episodic simulation when they are left to their own thoughts^33,34^. During rest, participants engage in higher order cognition with self-referential content such as: thinking about past events or simulating future events, reflecting about social interactions and current concerns, performing goal-directed planning and mental inferences about characteristics of self and others^35–37^, i.e. processes that require the integration of episodic information. Even though episodic simulation during presentation of empathic film clips and during rest might be two different forms of episodic simulation (directed towards a movie character vs. undriven cognitive processing), this procedure enabled us to compare resting state and task-evoked connectivity within the same participants. In real life, internal and external processing naturally fluctuate over time^38^. Therefore, studying both processing modes in combination represents an advance to previous studies of complex social cognition usually examining one state in isolation. To increase the power of the resting state analysis and to extend the results beyond the present sample of participants, we conducted identical analyses in an independent resting state data set of 198 participants, which was generously provided by Randy L. Buckner and is henceforth referred to as Cambridge sample, taken from the freely accessible database “1000 Functional Connectomes Project” (http://www.nitrc.org/projects/fcon_1000/)^39^.

Our previous study has shown that people are better able to empathize with others when they are given information concerning the context driving that person’s experiences. This suggests that people draw on prior memories when empathizing, but the mechanisms underlying this connection remain largely unexplored. The present study, in part using the same data of our previous study, investigates whether context information, that was shown to foster empathic concern towards a movie character, alters the connectivity between the TP and aHP. In addition, we tested whether the same connectivity pattern emerges in both resting state and task state. Unlike other processes, memory-based social cognition might represent a phenomenon in which people engage both during rest, and during task, when they are asked to^40^.

## Materials and Methods

### Participants

Twenty-eight healthy volunteers (mean age 29.85 ± 8.55 (sd), 1.61 (se), min = 19, max = 48 years (9 males, 19 females)) without history of any neurological or psychiatric disorder participated in the study. All participants were right-handed as assessed by the Edinburgh Handedness Inventory^41^ and had normal or corrected-to-normal vision. The study was divided in two experiments: The measurement of brain activity during rest, followed by the measurement of brain activity during task. Owing to head movements over voxel size and 3° during task, 2 participants had to be excluded leaving 26 subjects (17 females and 9 males, mean age 30.3 ± 8.7 years) for analysis of task-based effective connectivity. After a general screening for MR compatibility, participants were informed about the study and written consent was obtained. The study was approved by the ethics committee of the German Psychological Society. All procedures in this study were performed in accordance with the institutional guidelines and regulations. Participants either received course credit or were paid for their participation.

The Cambridge sample was used to replicate the functional connectivity findings during rest in an independent data sample. This sample consisted of 171 right-handers and 27 left-handers. Both samples (Berlin and Cambridge) were not significantly different for gender, but the Berlin sample was significantly older than the Cambridge sample (*t*(221) = 5.41, p < 0.001).

### Procedure

The participants were scanned using fMRI for 8 min of resting state (white fixation cross on black background, eyes open; Fig. 1A.1) followed by 52 min of task presenting 60 empathic film clips (Fig. 1A.2). The empathic film clips depicted a close-up of a character (30 males and 30 females) for 12 s with a sad facial expression and no mouth movements. The film clips were selected to elicit empathic concern for the movie character by showing a prolonged shot of the character’s face to focus the attention on his or her interior emotional experience^42^. A rating procedure ensured that the film clips were perceived as moderate on emotion scales (valence −0.36 ± 0.49 (scale -3 very unpleasant, 3 very pleasant), arousal 3.55 ± 0.51, sadness 4.06 ± 0.86, and compassion 3.80 ± 0.49 (scales 1 not at all, 7 very much). Post-scanning familiarity ratings on a scale from 1 (known) to 4 (unknown) confirmed that the film clips were not familiar to the participants (3.74 ± 0.19) (for details on the rating procedure, list of stimuli and technical details see Pehrs *et al*.^2^.

To investigate different neural activity induced by multisensory integration and contextual framing, the multisensory combination of the same visual information (i.e. empathic film clips) was systematically varied by music and context in a 2 × 3 design. The resulting 6 conditions are illustrated in Fig. 1A.2. Each condition included a 12 s empathic film clip preceded by a fixation cross with varying duration (4–8 s, mean 6.19 s). The first condition (Fig. 1A.2,1) showed a silent film clip without preceding context (“no context” condition = film only). The second condition (Fig. 1A.2,2) started with a neutral context followed by a silent film clip (“neutral context” condition = neutral context, film + no music). The neutral context described factual features of the character’s situation (e.g. sitting in a car, drinking a glass of water) and was used to control for general language processing. The third condition (Fig. 1A.2,3) started with a sad context followed by a silent film clip (“sad context” condition = sad context, film + no music). The sad context described people suffering from plights and was created based on the actual situation of the character in the movie. It described, for example, a recent loss of a loved person or getting informed about one’s infertility. The participants were informed that the sad context information provided the actual situation of the movie character and instructed to always refer to it when watching the film clip. The context information is used to measure episodic memory, because it stored in memory at the moment of film clip presentation. It is used by the participants as contextual framework to better understand the features of the scene (the semantic memory information, i.e. human faces in a contemplative mood). Conditions 4, 5, and 6 also arose from the 3 different context conditions (no/neutral/sad context), but included sad music played during the presentation of the film clip. Thus, the 6 conditions shown in Fig. 1A.2 are the result of all possible combinations of the factor context (no/neutral/sad) with the factor music (no/sad; Fig. 1A). The task design also featured a purely auditory control condition, in which sad music was presented during a 12 s fixation cross without the influence of meaningful visual or context information (Fig. 1A.2,7), resulting in 7 conditions in total. The 40 music pieces were presented randomly with the film clips to avoid multiple presentations of one film clip with a specific piece of music. The effect of music was analyzed in our previous study^2^. The present study focuses on the effect of context information on memory processing, specifically on episodic simulation in a social context. After every trial in each condition, a 5 s fixation cross was presented followed by 2 ratings, in which the participants used a tracking ball to rate their current emotional state in terms of “compassion” (“valence” for music only) and “being moved” on a 7-point Likert scale (1 not at all, 7 very much) for 8 s each. The results of the behavioral data have shown that the sad context significantly increased the experienced compassion of the participants towards the movie character compared to the neutral context and the no context condition (Fig. 1B.1). This behavioral effect was tracked on a neural level in the right TP and aHP bilaterally, as revealed by a parametric modulation analysis (Fig. 1B.2). This is important, because these results motivated the analyses of the present study focusing on the connectivity between TP and aHP and its change through context information that were shown to increase compassion. Ten trials per condition resulted in 70 trials presented in a fully randomized order during 51.67 min of continuous fMRI data acquisition. Participants were trained with 5 test trials inside the scanner. Further details can be found in Pehrs *et al*.^2^.

The task data of the same subjects were analyzed in our previous study to examine changes of effective connectivity in a multisensory integration network of emotion^2^. The present study addresses a different question, which is how changes of connectivity between brain regions that are key for different memory types support the use of episodic simulation in empathic concern. For this purpose, a different network structure was created consisting of bilateral TPs and aHPs and entered in an effective connectivity analysis. Another difference to the previous work is that brain connectivity was not only examined during task, but also during resting state using the same subjects.

### Data acquisition

Blood oxygen level-dependent (BOLD) fMRI was measured during rest and during task with a 3T Siemens (Erlangen, Germany) Tim Trio MRI scanner at the Dahlem Institute for Neuroimaging of Emotion (D.I.N.E.) using a 12-channel phased-array headcoil. The same whole-brain T2*-sensitive gradient-echo-planar imaging (EPI) sequence was applied in both experiments [TR 2000 ms; TE 30 ms; voxelsize 3 mm^3^; 240 scans; flipangle 70°; FOV 192 × 192 mm; matrix 64 × 64; 37 slices; 3 mm slice thickness; 0.6 mm gap]. Before resting state and task data acquisition, a high resolution T1-weighted structural image for registration of the functional data [TR 1900 ms; TE 2.52 ms; flipangle 9°; voxelsize 1 mm^3^; 176 sagittal slices; 1 mm slice] was taken. The whole scanning session lasted about 65 minutes.

The data of the Cambridge sample were acquired with a 3T Siemens scanner and a 12-channel vendor headcoil, while the participants had eyes open and saw a fixation cross during rest [TR 3000 ms, 47 slices, voxelsize 3 mm^3^, 119 scans].

### Analyses of fMRI data

#### Preprocessing

FMRI data was analyzed using the Statistical Parametric Mapping software package SPM8 (Wellcome Trust Centre for Neuroimaging, London, UK; http://www.fil.ion.ucl.ac.uk) implemented in MATLAB (version 2011a; The MathWorks Inc., Natick, MA, USA). The same preprocessing steps were applied to data from both resting state and task experiments and both samples (Berlin and Cambridge). Before preprocessing, the origin of the functional time series was set to the anterior commissure. Motion correction was performed using each subject’s mean image as a reference for realignment. The T1 image was coregistered to the mean functional image generated during realignment. The coregistered T1 images were segmented with the “New Segment” routine and normalized using DARTEL tools (Diffeomorphic anatomical registration through exponentiated lie algebra). The images were resliced with a 3 mm isotropic voxel size and smoothed with a 6 mm full-width at half maximum isotropic Gaussian kernel.

### Resting state functional connectivity

#### Seed-based functional connectivity

A resting state functional connectivity analysis was conducted to examine brain connectivity associated with a putative episodic simulation in the absence of task demands. This type of analysis is used to reveal intrinsic correlations between the signal of a seed region and the rest of the brain^43,44^. Individual time series were extracted from a 6 mm sphere around TP seeds bilaterally (Fig. 1B.1). We used two different sets of coordinates for timecourse-extraction for the effective connectivity analyses during task and the seed-based functional connectivity analysis during rest. This was done because it can not necessarily be assumed that connectivity during task and rest shows the same precise functional-anatomical pattern^45^. The left-hemisphere seed [−50 14–40; oriented in MNI space] was defined according to Andrews-Hanna *et al*.^46^. In this work, the authors have shown how different subsystems containing the TP and HP converge during rest to construct a mental scene based on episodic memories. A GLM containing a regressor of left TP-signal and ten regressors of no interest (cf. details below) was used to show commissural coupling and to define the seed coordinates as peak activation in the right TP [45 19–36]. Identical seed coordinates were used for analyses in the Cambridge and the Berlin sample.

Statistical analysis was performed using the GLM with a two-level approach^47^. On the first level, a GLM was performed containing the high-pass filtered (1/128 Hz) subject-specific signal timecourses of the seeds as regressors of interest. Nuisance covariates were added as regressors of no interest including the global signal of three separate brain compartments (all white-matter voxels (WM), all gray-matter voxels (GM), all CSF voxels and all out-of-brain voxels (OOB) as well as 6 head-motion parameters) resulting in a design matrix containing 12 regressors (2 regressors of interest (timecourses of right and left TP) and 10 regressors of no interest (WM, GM, CSF, OOB and 6 movement parameters). Three contrast images were created to determine functional connectivity of (i) the right TP seed, (ii) the left TP seed and (iii) both TP seeds together, which were taken to the second level (one-sample *t* tests) for a random-effects (RFX) analysis. The T-map corresponding to the first and second contrast images were used to report positive functional connectivity of the right and left TP separately at a significance level of p < 0.05 family wise error (FWE)-corrected across the whole brain and a cluster extent of 10 voxels. The T-map corresponding to the third contrast image (i.e. functional connectivity of both TP seeds together) was used for a region of interest (ROI) analysis and an analysis of laterality differences.

#### Region of interest analysis

A ROI analysis of bilateral HP was performed using anatomical masks taken from the Automated Anatomical Labeling^48^. Averaged individual parameter estimates within a 10 mm sphere around individual peaks within ROIs were extracted and represent the strength of positive functional correlations to the TPs.

#### Laterality Index

Laterality indices (LIs) are used as values to quantify relative differences in the engagement of the left versus right hemisphere regions. For analyses of LIs in the DMN, aHP and TP see SI Methods, SI Results, Figure S1.

### Task-based effective connectivity analysis

The univariate analysis of the task data that motivated the present effective connectivity analysis is described in Pehrs *et al*.^2^. In this previous work, a parametric modulation of the BOLD signal was applied to test for a linear relationship between regional signal changes depending on subjectively experienced empathic concern (i.e. compassion ratings). This analysis has shown that on a more lenient threshold (p < 0.001 uncorrected, cluster extent >5), activity in bilateral aHPs and TPs tracks empathic concern for the movie character (Fig. 1B.2, Table S3). It suggests that changes of connectivity between both regions, the aHP (associated with episodic memory) and the TP (associated with semantic memory) contribute to mnemonic integration (i.e. episodic simulation) during empathic responses, which is the question addressed by the present task-based effective connectivity analysis.

In contrast to analysis of resting state functional connectivity, analysis of task based effective connectivity can be used to test for changes of the coupling between brain regions as a function of experimental perturbations. For this purpose, dynamic causal modeling (DCM) version 10 as implemented in SPM8 (update r5236) was employed to examine the effects of context information and episodic simulation on connectivity between TP and HP (Fig. 1B.2)^49^.

DCM treats neural processing as dynamic input-state-output system^49,50^. The input enters the system by designed experimental conditions causing changes in neuronal responses (states), which are measured by changes in observed BOLD signals (output). A neuronal state is described by the endogenous and context-independent connections between regions (A matrix), the changes of these connections due to experimental perturbations (B matrix) (here neutral context (with and without music) and sad context (with and without music)) and the input in the system directly driving regional activity (C matrix) (here film clips (visual-V1) and music (auditory-STG)).

Model estimation as implemented in DCM takes prior densities to generate the posterior probabilities of the parameters using Bayesian inferences^49^, which are carried to classical statistical analysis for inferences about the strength and nature of the coupling effects (i.e. suppressing or enhancing).

Bayesian Model Selection (BMS) gives the so-called exceedance probabilities (EPs), the probability that one model is more likely than the other candidate models of the comparison. On family level, BMS compares sub-spaces of models and thus gives EPs for the entire families by taking a summation of posterior probabilities over family members^51^. Together, DCM and BMS allow conclusions about how activity in one region causes changes of activity in another region and how this pathway is changed by experimental perturbations (B matrix).

#### Modelspace

**Figure 2:**
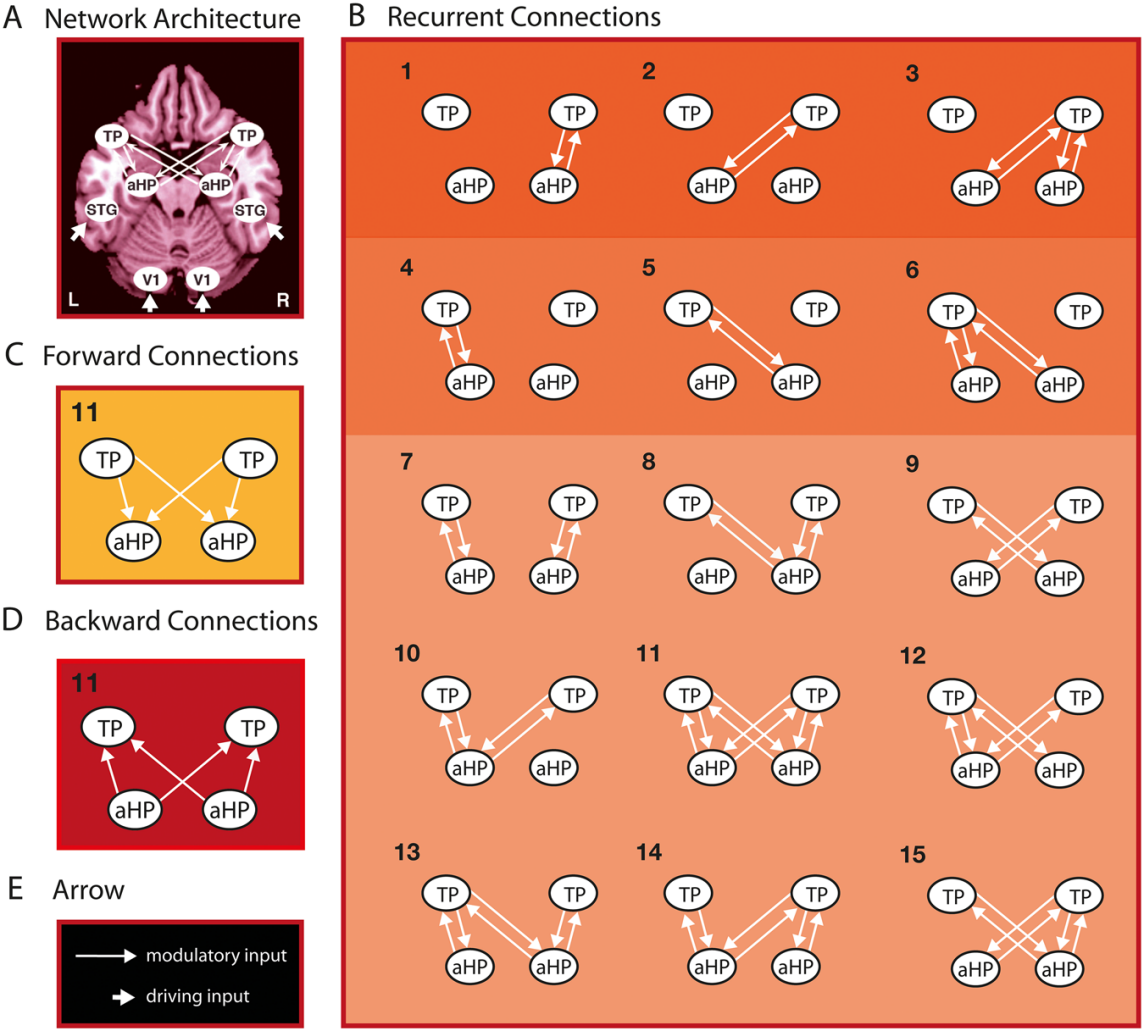
DCM Modelspace. (**A**) Basic model structure depicting all nodes of the bilateral symmetric network with driving input on V1 and STG (short arrows) and bilinear modulations between aHP and TP (long arrows). (**B**) Model-subspace showing all possible variations of bilinear modulations with recurrent connections. These 15 models were additionally modeled in a subspace with forward connections (**C**, from TP to aHP) and a subspace with backward connections (**D**, from aHP to TP) only. TP: temporal pole; aHP: anterior hippocampus; STG: superior temporal gyrus, V1: primary visual cortex.

A bilateral 8-area DCM was specified for all subjects with bidirectional endogenous connection between all regions (V1, STG, HP, TP) within and across hemispheres. Primary visual areas (V1 bilaterally) were defined as target regions for visual driving input during all conditions including film clip presentation (Fig. 1A.2, 1–6). Auditory cortices (superior temporal gyrus (STG) bilaterally) were defined as target regions for auditory driving input during all conditions with music (Fig. 1A.2, 4–7). A set of 15 models was created assuming all possible combinations of how the four context conditions (i.e. modulatory input, i.e. sad and neutral context conditions, each with and without music (Fig. 1C.2, Table S4)) could modulate connectivity of the four TP-HP connections within and across hemispheres (Fig. 2B). To note, we had no clear hypothesis on how the music would change HP-TP connectivity but included the neutral and sad context + music-conditions, because we were interested in how the context might change information propagation between these regions. To examine whether mnemonic integration is represented by information propagation to the HP, to the TP, or by reciprocal interactions of both brain regions, the 15 models were stratified in 3 model families. The first model family contained backward connections (aHP to TP, Fig. 2D), the second model family contained forward connections (TP to aHP, Fig. 2C) and the third model family contained recurrent connections (TP to aHP and aHP to TP, Fig. 2B). To define a relatively small model space, the direction (forward, backward, recurrent) was assumed to occur equally in both hemispheres and homotopic connections were omitted resulting in 45 models that were fitted and compared for each participant.

#### Timecourse extraction

Regional time series were extracted on the single-subject level using ROIs for HP and TP bilaterally. ROIs for extraction of time series were constructed using a combination of functional and anatomical criteria: second-level analysis clusters from a previous study^2^ revealed by the main effect of context for V1 and by the main effect of music for the superior temporal gyrus (STG) were masked with anatomical ROIs taken from the WFU Pick Atlas toolbox using the Automated Anatomic Labeling atlas (AAL)^48^. The STG was additionally masked with activation clusters from a previous study, in which we located an effect for multisensory integration of emotion^52^. For TP and HP, second-level analysis clusters revealed by the main effect of context^2^ were masked with ROIs taken from the AAL^48^. Each subject’s MNI coordinates of the highest t-value within these combined ROIs were surrounded with a sphere of 6 mm and used to extract the first eigenvariate based on a t-contrast including all conditions.

#### Bayesian Model Selection

Model comparison was implemented using random-effects (RFX) Bayesian model selection (BMS) to compute exceedance and posterior probabilities at the group level^53^. Exceedance probabilities (EPs) give the probability of a given model (or family of models) being more likely than any other model (or family of models; Penny *et al*.^51^) in the comparison, given the data. BMS was performed on two levels. First, we compared the model families and then we were looking for the best fitting model within the winning model family. For the optimal model, the subject-specific parameter estimates of the modulatory and driving inputs were entered into *t* tests at the group level using classical random effects analyses.

#### Data availability

The datasets generated during and/or analyzed during the current study are available from the corresponding author on reasonable request.

## Results

### Resting state functional connectivity

#### Seed-based functional connectivity

**Figure 3:**
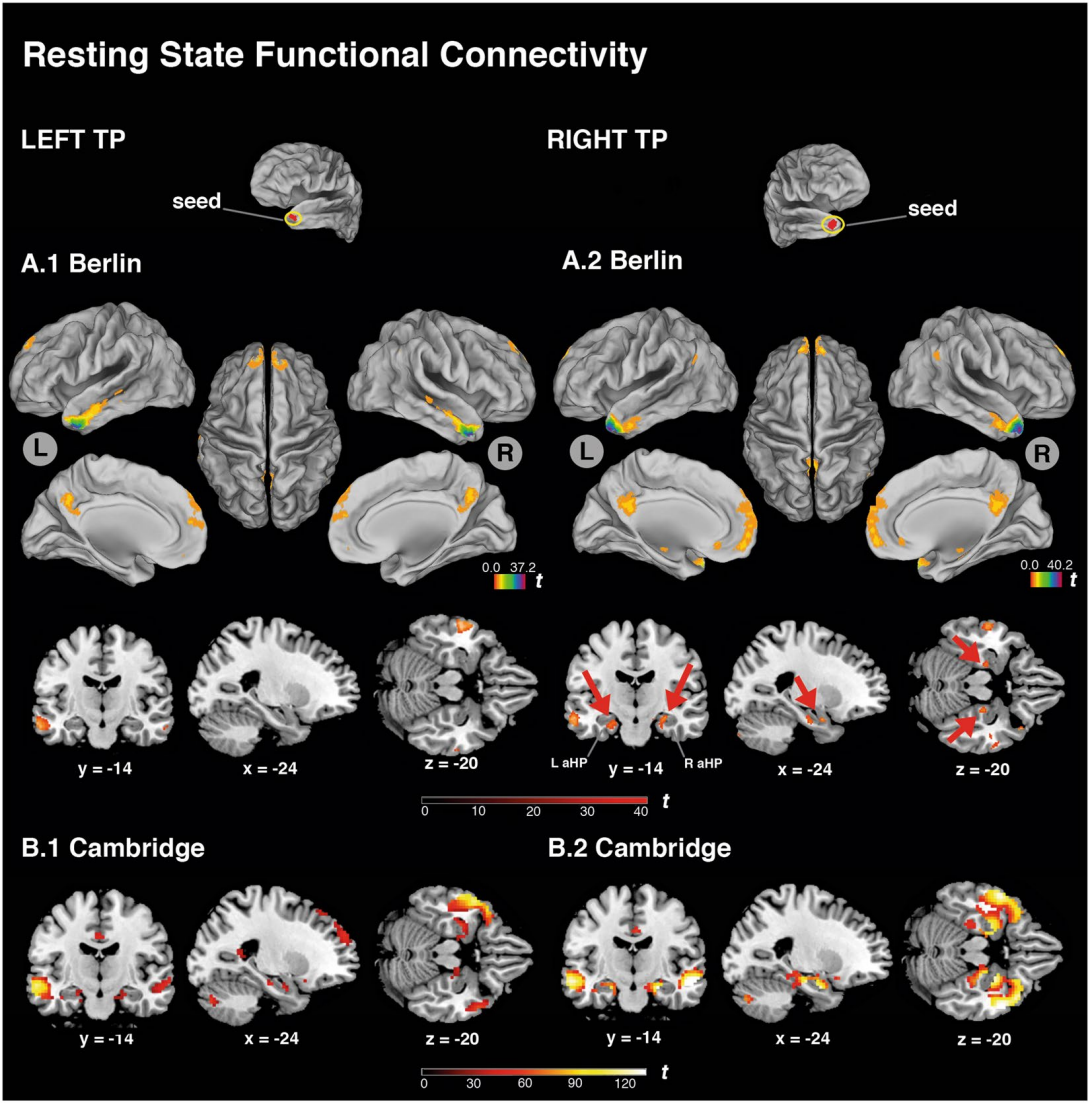
Resting state functional connectivity. (**A.1**) Connectivity pattern of left TP seed (see yellow ellipse on top left) superimposed on left (L) lateral, medial, dorsal, and right (R) lateral, medial, dorsal views and on coronal, sagittal and transaxial brain sections (below). (**A.2**) Connectivity pattern of the right TP seed; red arrows indicate functional connectivity to aHP bilaterally. (**B.1**) Same analyses in an independent data sample (Cambridge sample, n = 198) showing the connectivity pattern of left TP, superimposed on the same coronal, sagittal and transaxial brain sections. (**B.2**) Resting state functional connectivity of the right TP seed of the Cambridge sample. Coordinates on the bottom refer to MNI space. T-maps of resting-state connectivity are thresholded at p < 0.05, whole-brain FWE-corrected, cluster extent threshold k > 10 voxels. TP: temporal pole; aHP: anterior hippocampus.

The left TP showed resting state functional connectivity to core and extended DMN regions including medial prefrontal cortex, posterior cingulate cortex, left temporo-parietal junction, left superior temporal gyrus, middle temporal gyrus bilaterally, the right TP, but no functional connectivity to the HP (p < 0.05, whole-brain FWE-corrected, cluster extent >10 voxels; Fig. 3A.1, Table S1). The right TP, by contrast, showed resting state functional connectivity to bilateral aHP, and also to core regions of the DMN, namely the medial prefrontal cortex, posterior cingulate cortex, temporo-parietal junction bilaterally, and to regions of the extended DMN, such as the left middle temporal gyrus and the left TP (Fig. 3A.2, Table S1).

#### Region of interest analysis

**Figure 4:**
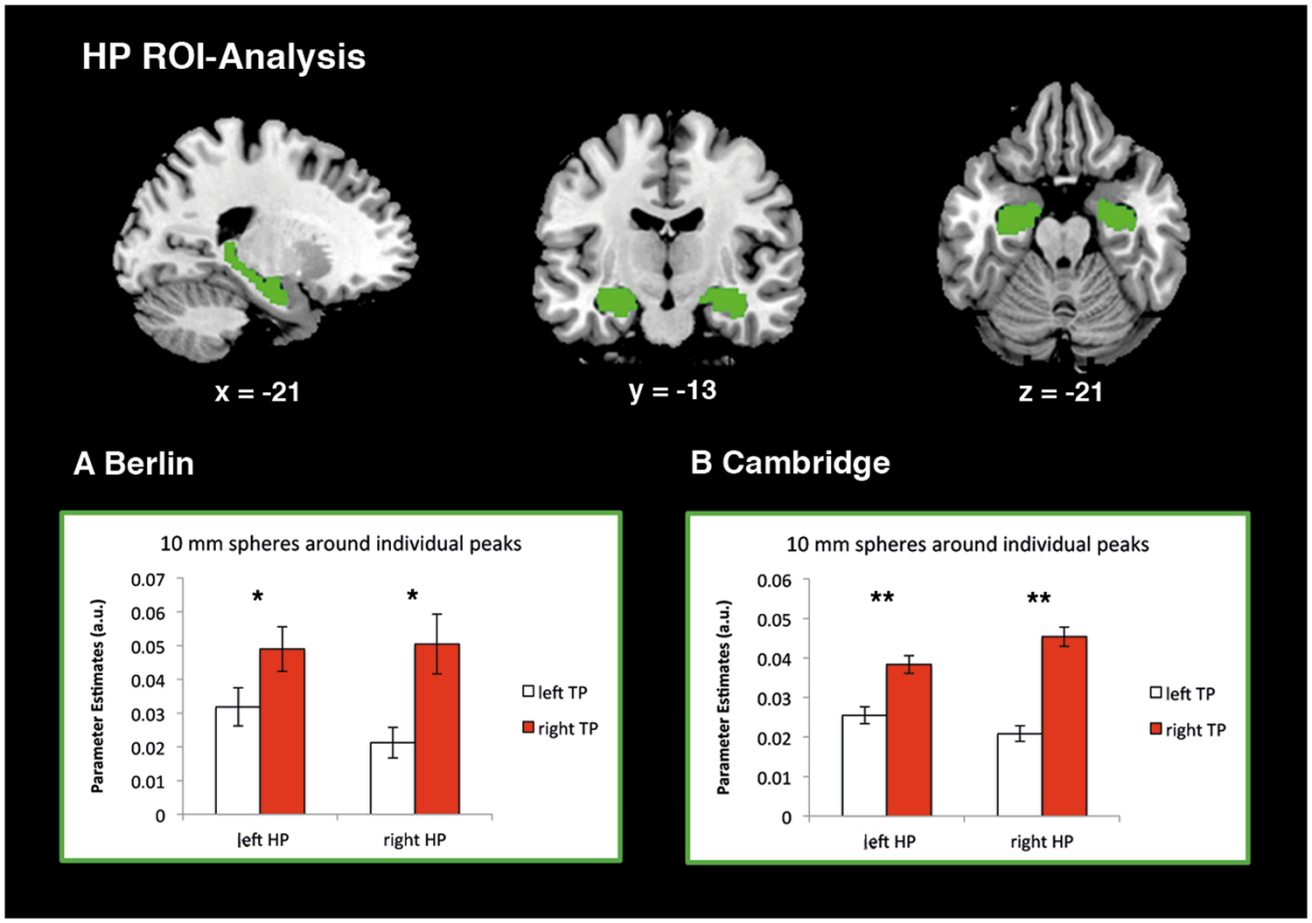
Region of interest (ROI) analysis of the HP. Sagittal (left), coronal (middle) transaxial (right) sections depicting HP-ROI (green) taken from the anatomic labeling atlas (AAL)^48^. Coordinates refer to MNI space. Bar plots depict averaged parameter estimates within a 10 mm sphere around individual peaks within left and right HP-ROIs of the (**A**) Berlin sample (n = 28) and (**B**) Cambridge sample (n = 198). Error bars represent standard error. TP: temporal pole; HP: hippocampus.

The HP-ROI analysis showed significantly stronger connectivity of the left and right HP to the right TP than to the left TP (p’s < 0.05) (Fig. 4A). This result is remarkable because ipsilateral connectivity is generally stronger than contralateral connectivity. Our findings emphasize the strong cross-hemispheric coupling of right TP and left HP during rest, likely supporting episodic simulation during self-relevant internal cognitive processes.

These results are supported by the same resting state functional connectivity and ROI analyses conducted in the Cambridge sample (n = 198). This extends the results of the functional connectivity analyses beyond the present study sample. In the Cambridge sample, resting state functional connectivity to the HP was more pronounced for the right than for the left TP (Fig. 3B.2, Table S2). ROI analyses showed significantly higher connectivity of bilateral HP to the right TP than to the left TP (p’s < 0.001) (Fig. 4B).

### Task based effective connectivity

**Figure 5:**
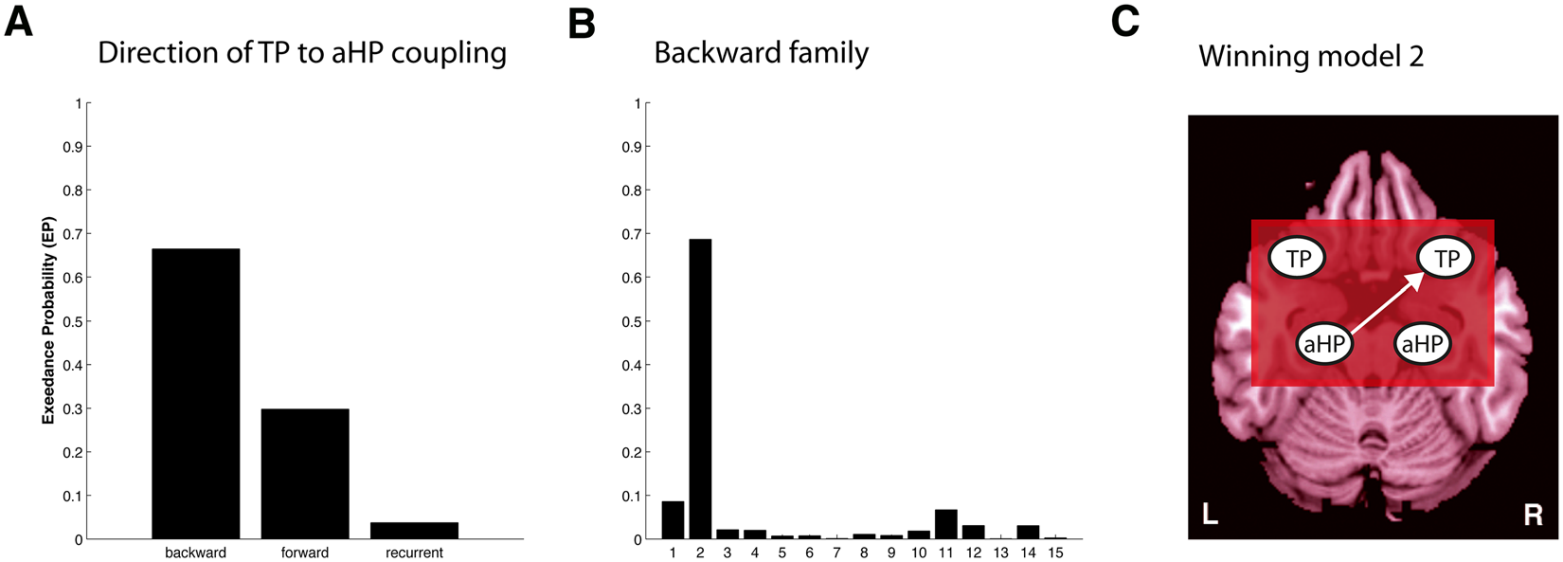
Results of Bayesian model selection (BMS). BMS was performed on two levels. (**A**) Family selection of backward, forward and recurrent TP to aHP connectivity and (**B**) model selection for the backward model-subspace containing 15 models, with all possible combinations of context modulation on aHP to TP connections. (**C**) The winning model, model 2, shows effective backward connectivity from left aHP to right TP. Exceedance probabilities (EPs) are reported as a measure of relative model fit. TP: temporal pole; aHP: anterior hippocampus.

The aim of the effective connectivity analysis was to test whether context information changes the neural coupling between the HP and the TP. Random-effects family-based inference comparing backward (aHP to TP), forward (TP to aHP) and recurrent families (aHP to TP and TP to aHP) revealed that the backward family outperformed the forward and the recurrent families with an exceedance probability (EP) of 66.44% (Fig. 5A). The subsequent model comparison within the backward family revealed that the most plausible model contains backward projections from left aHP to right TP with an EP of 68.56%, compared with EP’s of ≤8.6% for all of the 14 models of the comparison (Fig. 5B). In correspondence with the strong coupling of right TP and left aHP during rest, the winning model structure indicates that context information to the film clip increases neural propagation from left aHP to right TP (Fig. 5C). This shows that the left aHP associates the episodic context information with semantic information during empathic film clip presentation by modifying its (mnemonic) inputs to the right TP. The nature of how a neural coupling between two regions is changed by experimental manipulations (i.e. whether it is inhibitory or enhancing), can be detected by statistical inferences on subject-specific parameter estimates. In correspondence with our hypotheses, we would have expected significant differences between the neutral and the sad context conditions. This was not found in the present study. For inspections on parameter estimates see SI Results, Tables S4 and S5.

## Discussion

This study investigates the role of episodic simulation in empathic concern. Our previous study showed that the more background information people receive about another person, the better they are able to put themselves into their shoes, with the right temporal pole playing a critical role in this process^2^. The present study links these findings more explicitly to memory processing by showing that the temporal pole (TP) likely contributes to empathic concern via *episodic simulation*. Episodic simulation requires the integration of different memory types. During mnemonic integration, neural information needs to be exchanged between regions that are associated with different memory types, such as the TP for semantic memory and the anterior hippocampus (aHP) for episodic memory. Our study reveals a crossed-hemispheric mechanism that may support episodic simulation, assuming that the right TP integrates episodic information from the left aHP during both resting state and task state. Empathic deficits in psychiatric disorders, such as autism spectrum disorder, are discussed regarding putative impairments of this mechanism.

### The role of the right temporal pole in emotion processing

Crossed-hemispheric connectivity from left HP to right TP maps onto previous findings of laterality differences for these regions. The left HP is particularly involved in processing of episodic information (for a review see Svoboda *et al*.^17^). Recent evidence also indicates a functional segregation along the longitudinal axis, in which the aHP is predominantly involved in social and emotional memory^54,55^, whereas the posterior HP is engaged in more cognitive functions such as spatial memory^56,57^. This is also supported by increased activity in the aHP as a function of vividness, emotionality, and personal significance of episodic memory contents^58,59^. Correspondingly, timecourse extraction for DCM analysis was located in the anterior portion of the HP, in line with a linear increase of activity in this region with compassion ratings of the participants (Fig. 1B.2, Table S6).

The right TP was previously associated with semantic processing of emotional content^60,61^. This is the first study to link these laterality differences to memory processes. We argue that the right TP is more strongly involved in emotion processing because of its function to integrate episodic, emotional memory. The left TP, by contrast, might be rather engaged in tasks requiring language processing. Indeed, the right TP is associated with retrieval of familiar or famous faces^62^, reliably responds to empathy and theory of mind tasks^63^, for the processing of social vs. animal concepts^26^, and for personally relevant compared with non-personal memories^58^, i.e. tasks that demand the integration of episodic memory. The left TP, however, was found to be involved in verbal processing like learned name retrieval^64^ or processing of short narratives^65,66^. The left TP also shows enhanced functional connectivity to the temporo-sylvian language network supporting its specialization for verbal material^67^.

### Lateral integration as a marker for social cognition

The neural mechanism is specifically characterized by its information propagation across the two brain hemispheres from left aHP to right TP, which could be of advantage in an evolutionary perspective. Hemispheric specialization and commissural integration is generally proposed to be an evolutionary advantage that allows a more efficient allocation of brain resources. In this way, existing brain function is retained, but novel brain function becomes enabled through lateral specialization^68^. Language processing, for example, is in its complexity unique to the human species and shows lateral specialization to the left hemisphere^69^. Advanced higher-order social cognition, such as episodic simulation, might similarly have developed through lateral specialization. A cross-talk of left aHP, associated with episodic memory, and right TP, associated with semantic memory, might in this respect foster mnemonic integration. A recent diffusion tensor imaging study in a sample of 949 adolescents has shown that female brains have a greater interhemispheric connectivity than male brains^70^. The authors linked these findings to sex differences in human behavior showing that females have superior social memory and social cognition skills. This may provide indirect evidence for the importance of interhemispheric communication for complex social cognition, in line with the results of our study.

### Intrinsic vs. extrinsic brain connectivity

The analyses in the present study show evidence for the crossed-hemispheric mechanism not only during a social cognition task, but also during rest when people let their mind wander. Despite originally discussed to engage antagonistic brain networks, mounting evidence indicates that intrinsic and extrinsic processing modes show overlapping patterns of brain connectivity. Cole *et al*.^71^ compared functional connectivity during rest and multiple tasks (social, emotional, n-back, motor etc.) and revealed a striking overlap of network activity irrespective of the type of task. Recent studies have also shown that tasks requiring constructive memory processing, specifically in a social context^37,72^, also engage the DMN (^29,30,73,74^; for a review see Andrews-Hanna *et al*.^75^). Social tasks engaging the DMN were targeting processes like social working memory^76^, processing of self and others^77,78^, and theory of mind^74,79^. To the best of our knowledge, only one other study directly compared resting state and task-evoked effective connectivity using the same set of participants and a hand movement task^80^. The authors found a weak overlap of brain connectivity and suggested to use methods of intrinsic and extrinsic connectivity as complementary measures.

Our study, on the contrary, shows similar brain connectivity during rest and during task, challenging the traditional dichotomous conceptualization of spontaneous and evoked activity. We argue that this is due to the social nature of our task implying similar cognitive processes that occur during rest. For a discussion on how attention demands shape the reciprocal interplay of processing modes in social contexts see SI Discussion.

### Socio-emotional problems in psychiatric disorders and memory processing

Our results show that the integration of episodic memory helps to appropriately empathize with a social target, which is an important ability for social functioning. Impairments in social and emotional functioning are a frequent symptom of a variety of psychiatric disorders. Nonetheless, research linking memory processing and social functioning in clinical populations is sparse, despite evidence pointing to deficient mnemonic integration.

First, psychological and socio-emotional problems are particularly pronounced in patients with damage to the right TP^81,82^. Second, a recent study has shown reduced hippocampal-temporal pole functional connectivity as a biomarker for diagnoses of social anxiety disorder. This feature disappeared after 8 weeks of treatment with the selective serotonin reuptake inhibitor (SSRI) paroxetine, concomitant with a decrease of symptom severity^83^. Third, children with ASD show deficits in integrating episodic memory in the narration of an autobiographic study^84^ as well as deficits in episodic but not semantic memory using a recognition memory task^85^. The present data suggest the restricted ability to read others person’s minds and imagine their thoughts and feelings, which is a core symptom of ASD^86^, might be associated with deficits in episodic simulation. Prompting individuals to relate to context information when trying to understand others may be used for therapeutic benefits.

## Limitations

Semantic and episodic memory processing involve large-scale brain networks including additional brain regions beyond TP and HP. Based on the literature^17,32^ and the results of a previous study^2^, the present work focuses on the connectivity between two brain regions and not on the connectivity within large-scale brain networks. With this approach, we were able to reveal a neural mechanism underlying episodic simulation. Nonetheless, we are encouraged to develop additional hypotheses, such as a possible control function of ventrolateral prefrontal cortex on aHP to TP coupling^87^.

Although episodic simulation is strongly supported by the results and by the literature, other experimental designs with systematic variations of AEM and SM should address mnemonic integration during episodic simulation. These should be able to dissociate the neural memory and empathy network and their interactions including explicit memory tests.

However, note that patients with Alzheimer’s disease and neurodegenerative changes in medial temporal and anterior temporal regions, show impairment in using episodic memory for the simulation of future events^88^, strongly suggesting that activity in HP and TP is critical for episodic simulation.

## Conclusions and Outlook

This study shows that memory processes promote empathic concern. Context information enables individuals to fully grasp the current emotional state of others and to feel more compassionate towards them. The required mnemonic integration involves changes of connectivity between key structures specialized for different memory types. We show that the right TP integrates semantic memory with episodic memory from the left aHP while context information triggers people to episodically simulate the mind of another individual. With regard to laterality differences of the TP, we put forward the idea that the right TP plays a critical role in emotion processing because of its function to integrate episodic emotional information. The same connectivity profile was shown during rest, indicating how episodic information is integrated when constructing a mental scene during unconstrained thought.

Another facet of the episodic simulation hypothesis is that people use own experiences to simulate other’s thoughts and feelings^89,90^. Retrieving similar situations from one’s own history, namely self-referential memories, may be of additional help to understand and feel with others^91,92^.

In addition, neural propagation across hemispheres, between left HP and right TP is most likely conveyed via the anterior commissure^93^. Future diffusion tensor imaging studies could examine how different fiber strength in the anterior commissure modulates the capacity to engage in episodic simulation.

## Electronic supplementary material

Supplementary Info
