# Supplementary material for: Hippocampal-Temporopolar Connectivity Contributes to Episodic Simulation During Social Cognition
Source: Sci Rep. 2018 Jun 20;8:9409. doi: 10.1038/s41598-018-24557-y (PMC6010422; doi:10.1038/s41598-018-24557-y)
Supplement: Supplementary file 1 — Supplementary Info [file 41598_2018_24557_MOESM1_ESM.pdf]

Supplemental Information

Hippocampal-Temporopolar Connectivity contributes to Episodic Simulation during Social Cognition

Corinna Pehrs, Jamil Zaki, Liila Taruffi, Lars Kuchinke, Stefan Koelsch

SI Methods

Laterality Index. Laterality indices (LIs) are used as values to quantify relative differences in the engagement of the left versus right hemisphere regions. They provide a numerical quantification of lateralization and are preferable to simple visual inspection.

To account for thresholding effects, the LI toolbox was used (version 1.2) [1,2], which generates weighted mean LIs (LI_Ws) iteratively exploring increasing thresholds using a multithresholded bootstrap-method. Individual weighted LIs favor regional activations, which show higher correlation with the task (in our case functional connectivity with bilateral TP), such that they receive stronger impact on the resulting LI values. In the present study we report the group mean of individual LI_Ws in three ROIs (Fig. S1). These were created using activations of bilateral resting state functional connectivity patterns with the study's specific threshold of $p < 0.05$, FWE-corrected, and cluster extent of 10 voxels. Masks covering the posterior cingulate cortex, medial prefrontal cortex, temporoparietal junction, HP, TP, middle temporal gyrus in both hemispheres were flipped, combined and binarized, resulting in a symmetric bilateral default mode network (DNM) mask. Masks for aHP and TP were created using a combination of functional and anatomical criteria: activation clusters from resting state functional connectivity analysis were masked with anatomical regions taken from the automatic anatomic labeling atlas [3] and subsequently flipped, combined, binarized and entered in LI-analyses (Fig. S1).

LI_W values lie on a continuum between +1 (only left) and -1 (only right). The threshold for hemispheric lateralization was > ±0.2, as suggested previously [4].

SI Results

Laterality index. Taking functional connectivity maps of bilateral TPs (i.e. contrast image derived from both TP seeds together), weighted laterality indices (LI_Ws) revealed a right lateralization of functional connectivity to the DMN [-0.36 ± 0.02(se)], a right lateralization of functional connectivity to the TPs [LI_W mean -0.38 ± 0.02(se)], but a left lateralization of functional connectivity to the aHP [0.25 ± 0.08(se)] (Figure 4). This corroborates the specific cross-talk between

the right TP and left HP. Consistently, a right lateralization of the DMN was previously reported to be associated with memory-based construction [5].

DCM – Inspection on parameter estimates. The posterior distributions of the estimated parameters can be used to test hypotheses about connection strengths and its modulation quantitatively. The endogenous connections in DCM models reflect the overall connectivity between two regions within an experiment, and modulatory parameters reflect changes of the endogenous connection strength as a function of an experimental manipulation, here neutral/sad context (see Materials and Methods). Inspection on parameter estimates of the winning model revealed that the endogenous connection from left aHP to right TP (0.031, 0.02(se)) is enhanced about 0.08 Hz by sad context, 0.17 Hz by neutral context, 0.05 Hz by sad context and music, and 0.04 Hz by neutral context and music. There is a considerable inter-individual difference for parameter estimates and the nature of the effect (Table S4, Table S5). Future studies should determine when aHP's influence on TP is suppressing or facilitating using social memory tasks specifically tailored to address this question.

SI Discussion

Reciprocal interplay of processing modes. Real-life social interactions and naturalistic social cognition tasks are an excellent case of mutual cooperation of internal and external processing modes. Social interactions demand the combination of external socio-emotional cues like language, facial expressions and biological motion with internal socio-emotional cues like attending to one's own feelings and drawing inferences on others' mental states. Therefore, internally and externally driven cognition constantly interact and mutually cooperate in a social context. Dixon et al. [6] suggested that a reciprocal interplay of processing modes is mediated by processing demands. Co-occurrence and co-operation between internally and externally oriented modes is possible to the extent that both require a balanced amount of cognitive load with minimal interference [6]. This might specifically apply to social interactions. Social interactions require a variety of interdependent cognitive functions, rapidly fluctuating between internally and externally directed processing. Instead of studying task-evoked brain activity in isolation, social cognition research should develop tasks that directly target different functional states to examine the variability of spatial networks along the internal-external continuum.

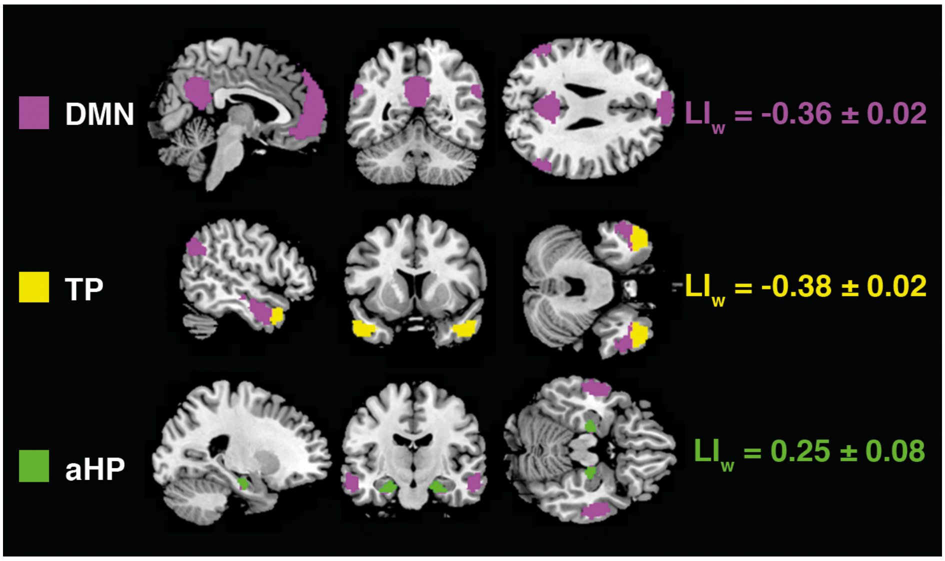

Fig. S1. Laterality index (LI) analysis. ROIs covering the DMN (top, violet), TP (middle, yellow) and aHP (bottom, green) with weighted LI group means on the right and standard error. For LIw-calculations were applied to resting state functional connectivity patterns of bilateral TP. LIs vary between -1 (right lateralization) and +1 (left lateralization). To note, green and yellow colored ROIs were also part of the DMN mask (violet). DMN: default mode network, TP: temporal pole, aHP: anterior hippocampus.

Table S1. Positive Resting State Functional Connectivity - Berlin dataset (n = 28).

Region	Brodmann areas	x	y	z	Cluster size	*t* value
**Seed left TP [-50 14 -40][1]**						
L. temporal pole	38	-48	9	-38	1288	43.97
L. middle temporal gyrus	21	-58	-6	-27		
L. middle temporal gyrus	21	-64	-13	-17		
Posterior cingulate cortex	7	0	-58	34	851	9.95
R. Cerebellum Crus I/II		36	-81	-38	750	9.80
R. Cerebellum Crus I/II		27	-84	-36		
R. Cerebellum Crus I/II		21	-75	-30		
R. temporal pole	38	57	-1	-29	101	9.16
R. middle temporal gyrus	21	62	-4	-23		
Medial prefrontal cortex	9, 32	0	60	24	1107	9.15
L. dorsal medial prefrontal cortex		-9	53	40		
L. dorsal medial prefrontal cortex	8	-16	53	37		
L. Cerebellum Crus I/II		-40	-78	-39	92	8.97
Medial prefrontal cortex	11, 24, 25, 32	0	54	-12	164	7.89
R. medial prefrontal cortex	10, 24	6	48	4	44	7.83

L. superior temporal gyrus	22, 21	-66	-33	3	40	7.59
R. middle temporal gyrus		63	-15	-21	13	7.55
L. middle temporal gyrus	21	-52	-30	-5	91	7.46
L. tempoparietal junction	21,22	-52	-66	39	122	7.39
**Seed right TP [45 19 -36][1]**						
R. temporal pole	38	45	18	-36	1188	70.26
R. temporal pole	21	56	8	-32		
R. temporal pole	47	44	21	-20		
medial prefrontal cortex	11, 24, 25, 32, 9, 32	0	56	25	3104	11.28
R. dorsal medial prefrontal cortex	8	12	56	36		
Ventral medial prefrontal cortex		2	60	-9		
L. temporal pole	38	-38	9	-32	576	10.08
L. temporal pole	21	-54	6	-27		
Posterior cingulate cortex	23,31	0	-52	28	1369	10.06
Posterior cingulate cortex/Precuneus	31	10	-48	36		
L. Cerebellum Crus I/II		-40	-78	-38	116	9.80
L. Cerebellum Crus I/II		-27	-85	-39		
R. Cerebellum Crus I/II		40	-78	-39	107	9.70
R. Cerebellum Crus I/II		30	-85	-36		
L. middle temporal gyrus	20	-58	-10	-24	354	9.45
L. middle temporal gyrus		-60	-15	-15		
R. temporoparietal junction	39	60	-54	27	161	8.94
R. temporoparietal junction		55	-57	33		
L. temporoparietal junction	21, 22	-56	-60	28	159	8.36
R. amygdala		24	0	-23	36	8.18
R. anterior hippocampus	20, 36	27	-16	-17	116	8.01
L. anterior hippocampus	20, 36	-26	-12	-24	97	7.58

Note: Reported are regions that show a significant activation at $p < 0.05$ whole-brain FWE-corrected, cluster extent > 10 voxels. Brodmann areas list approximate locations for reference. Subpeaks > 8mm from the main peak in each cluster are listed. [1]The timecourses were extracted within a 6mm sphere around MNI coordinates.

Table S2. Positive Resting State Functional Connectivity - Cambridge dataset (n = 198).

**Region**	**Brodmann areas**	**x**	**y**	**z**	**Cluster size**	***t* value**
**Seed left TP [-50 14 -40][1]**						
L. temporal pole	38	-48	12	-36	1196	139.18
L. middle temporal gyrus	21	-60	-18	-9		15.79
L. middle temporal gyrus	21	-60	-39	0		15.63

L. medial prefrontal cortex	8	-9	54	36	1817	17.37
L. medial prefrontal cortex	6	-9	36	54		16.42
L. medial prefrontal cortex	8	-6	45	48		15.56
L. temporoparietal junction	21,22	-45	-60	27	545	16.40
Posterior cingulate cortex	7	-6	-48	33	517	13.53
L. middle frontal gyrus	8	-42	15	51	190	12.14
R. Cerebellum Crus I		27	-78	-33	101	12.07
R. temporal pole	38	54	12	-27	281	11.63
R. middle temporal gyrus	21	66	-30	-3		9.59
R. middle temporal gyrus	21	60	-9	-12		9.28
R. temporoparietal junction	39	57	-60	30	178	10.87
R. temporoparietal junction	40	48	-54	30		8.89
L. Cerebellum Crus I		-27	-78	-33	22	9.26
L. Thalamus	30	-9	-33	6	227	8.83
R. Frontal Pole		48	36	-12	15	8.79
Cingulate gyrus		0	-18	36	26	7.27
R. Cerebellum, IX		6	-54	-48	22	6.84
L. Cerebellum, IX		-3	-54	-48		5.96
R. Hippocampus		15	-12	-21	23	6.12
R. Hippocampus		24	-15	-18		5.89
**Seed right TP [45 19 -36][1]**						
R. temporal pole	38	45	18	-33	2229	89.16
R. middle temporal gyrus	21	57	3	-21		16.63
R. middle temporal gyrus	22	60	-12	-12		16.60
L. temporal pole	38	-42	21	-30	1008	21.62
L. temporal pole	38	-51	9	-27		17.40
L. hippocampus		-27	-3	-27		14.03
R. medial prefrontal cortex	9	6	54	24	1630	14.86
R. medial prefrontal cortex	10	3	51	-9		12.19
R. Frontal Pole	8	12	45	51		10.43
L. temporoparietal junction	21,22	-45	-60	27	312	10.58
L. temporoparietal junction	21,22	-54	-66	27		10.06
L. Cerebellum Crus I/II		-24	-78	-36	20	8.12
Cingulate gyrus	24	3	-15	36	12	6.30
Anterior cingulate cortex	25	0	6	-3	10	6.13

Note: Reported are regions that show a significant activation at $p < 0.05$ whole-brain FWE-corrected, cluster extent > 10 voxels. Brodmann areas list approximate locations for reference. [1]The timecourses were extracted within a 6mm sphere around MNI coordinates.

Table S3. Parametric BOLD effects of compassion ratings.

Region	Brodmann area	x	y	z	Cluster size	t value
L. superior frontal gyrus	B8	-9	18	51	10	5.35
R. hippocampus		30	-9	-15	21	5.22
R. amygdala		30	0	-21		4.92
R. temporal pole	38	51	12	-27	62	5.04
R. temporal pole	38	54	6	-18		4.81
R. premotor cortex	6	48	0	57	18	4.91
R. premotor cortex	6	54	0	51		4.86
R. superior temporal gyrus	22	48	-33	3	54	4.86
R. superior temporal gyrus	22	48	-21	-3		4.41
R. middle temporal gyrus	22	57	-24	0		3.52
L. occipital pole	17	-18	-99	18	34	4.86
L. inferior frontal gyrus	45	-42	30	-3	16	4.81
L. hippocampus		-30	-9	-18	9	4.61
R. mamillary body		6	-6	-15	16	4.57
R. brainstem		3	-15	-12		4.19
L. middle frontal gyrus	44	-33	0	42	26	4.45
L. premotor cortex	6	-36	-6	48		3.96
L. temporal pole		-48	3	-24	7	4.38
R. superior temporal gyrus		57	-36	15	18	4.30
R. supramarginal gyrus		63	-39	9		3.86
L. Crus II		-21	-78	-36	8	4.25
R. premotor cortex	6	6	15	63	5	3.98

Note: Reported are regions that show a significant activation at $p < 0.001$ uncorrected, cluster extent > 5 voxels. Brodmann areas list approximate locations for reference. Two participants were excluded due to missing values in behavioral data leaving 24 subjects for this analysis (cf. Pehrs et al., 2015).

Table S4. DCM Parameters (B Matrix). Mean connectivity parameters for the winning model 2 (cf. Figure 5) comprising bilinear modulations of context and music (separated for pos and neg values) on the connection from left aHP to right TP with standard error (se) in brackets. *T*-tests were performed and *t* and *P* values are reported.

	pos/neg	mean (SE)	*t* value	*P* value
**Modulatory input on L. aHP to R. TP**				
Sad context, film + music		0.054 (0.14)	0.38	0.70
	16 pos	0.47 (0.11)	4.30	0.001*
	10 neg	-0.62 (0.16)	3.69	0.005*
Sad context, film + no music		0.087 (0.14)	0.59	0.56
	18 pos	0.48 (0.07)	6.30	<0.001**
	8 neg	-0.79 (0.25)	3.18	0.01*
Neutral context, film + music		0.04 (0.14)	0.28	0.78
	14 pos	0.55 (0.1)	5.16	<0.001**
	12 neg	-0.55 (0.16)	3.44	0.005*
Neutral context film + no music		0.175 (0.12)	1.45	0.15
	18 pos	0.47 (0.08)	5.27	<0.001**
	8 neg	-0.49 (0.18)	2.71	0.03*

Note: ** $p < 0.001$, * $p < 0.05$, Bonferroni-corrected for multiple comparisons.

Table S5. DCM Parameters (C Matrix). Mean connectivity parameters for the winning model 2 (Figure 5) comprising driving input with standard error (se) in brackets. *T*-tests were performed and *t* and *P* values are reported.

		mean (SE)	*t* value	*P* value
**Driving input**				
Sad context, film + music	⇒ L. V1	0.06 (0.02)	2.35	0.027*
	⇒ R. V1	0.1 (0.04)	2.57	0.016*
	⇒ L. STG	0.39 (0.04)	8.75	<0.001**
	⇒ R. STG	0.41 (0.04)	9.73	<0.001**
Neutral context, film + music	⇒ L. V1	0.08 (0.03)	2.39	0.024*
	⇒ R. V1	0.11 (0.04)	2.55	0.017*
	⇒ L. STG	0.39 (0.04)	8.86	<0.001**
	⇒ R. STG	0.44 (0.04)	9.53	<0.001**
Sad context, film + no music	⇒ L. V1	0.18 (0.04)	4.42	<0.001**
	⇒ R. V1	0.18 (0.05)	3.26	0.003*
Neutral context film + no music	⇒ L. V1	0.19 (0.04)	4.75	<0.001**
	⇒ R. V1	0.18 (0.05)	3.14	0.004*

Note: ** $p < 0.001$, * $p < 0.05$, Bonferroni-corrected for multiple comparisons.

Table S6. MNI coordinates for DCM timecourse extraction within aHP. The coordinates show that peak activations of the individuals for DCM-analysis were located in the anterior part of the HP.

**Left HP**			**Right HP**		
x	y	z	x	y	z
-21	-12	-15	24	-15	-15
-21	-9	-21	21	-9	-15
-18	-12	-18	21	-6	-18
-24	-15	-15	21	-6	-15
-21	-21	-18	24	-21	-18
-21	-9	-15	21	-6	-15
-21	-12	-18	21	-6	-18
-21	-9	-21	21	-6	-18
-24	-9	-18	21	-6	-18
-21	-9	-21	21	-6	-15
-21	-18	-18	24	-18	-18
-21	-18	-15	24	-15	-18
-21	-15	-15	21	-6	-15
-24	-12	-15	21	-9	-18
-21	-21	-18	21	-6	-15
-27	-9	-18	21	-6	-15
-21	-9	-15	21	-6	-18
-21	-12	-15	21	-9	-15
-21	-12	-12	21	-18	-18
-21	-9	-15	21	-6	-18
-24	-6	-24	30	-15	-24
-21	-9	-15	21	-6	-15
-21	-9	-15	21	-9	-12
-24	-9	-12	21	-6	-15
-21	-15	-15	21	-9	-15
-33	-12	-15	21	-6	-15

Note: MNI – Brain space of the Montreal Neurological Institute (average of 152 normal MRI scans).

1. Wilke, M. & Schmithorst, V. J. A combined bootstrap/histogram analysis approach for computing a lateralization index from neuroimaging data. *Neuroimage* **33,** 522–530 (2006).

2. Wilke, M. & Lidzba, K. LI-tool: A new toolbox to assess lateralization in functional MR-data. *J. Neurosci. Methods* **163,** 128–136 (2007).

3. Tzourio-Mazoyer, N. *et al.* Automated anatomical labeling of activations in

SPM using a macroscopic anatomical parcellation of the MNI MRI single-subject brain. *Neuroimage* **15,** 273–289 (2002).

4. Seghier, M. L. Laterality index in functional MRI: methodological issues. *Magn. Reson. Imaging* **26,** 594–601 (2008).

5. O'Callaghan, C., Shine, J. M., Lewis, S. J. G., Andrews-Hanna, J. R. & Irish, M. Shaped by our thoughts - A new task to assess spontaneous cognition and its associated neural correlates in the default network. *Brain Cogn.* **93,** 1–10 (2015).

6. Dixon, M. L., Fox, K. C. R. & Christoff, K. A framework for understanding the relationship between externally and internally directed cognition. *Neuropsychologia* **62,** 321–330 (2014).

7. Pehrs, C. *et al.* The temporal pole top-down modulates the ventral visual stream during social cognition. *Cereb. Cortex* **27,** 777–792 (2017).
